# Building a trusted framework for uncertainty assessment in rare diseases: suggestions for improvement (Response to “TRUST4RD: tool for reducing uncertainties in the evidence generation for specialised treatments for rare diseases”)

**DOI:** 10.1186/s13023-020-01666-4

**Published:** 2021-02-01

**Authors:** Sabine E. Grimm, Xavier Pouwels, Bram L. T. Ramaekers, Ben Wijnen, Saskia Knies, Janneke Grutters, Manuela A. Joore

**Affiliations:** 1 Department of Clinical Epidemiology and Medical Technology Assessment, School for Public Health and Primary Care (CAPHRI), Maastricht University Medical Centre, P. Debyelaan 25, PO Box 5800, 6202 AZ Maastricht, Netherlands; 2 University of Twente, Hallenweg 5, 7522 NH Enschede, Netherlands; 3 Zorginstituut Nederland, Eekholt 4, 1112 XH Diemen, Netherlands; 4 Department for Health Evidence, Radboud University Medical Centre, Post 133, PO Box 9101, 6500 HB Nijmegen, Netherlands

**Keywords:** Health technology assessment, Uncertainty, Health economics, Orphan diseases

## Abstract

The aim of this letter to the editor is to provide a comprehensive summary of uncertainty assessment in Health Technology Assessment, with a focus on transferability to the setting of rare diseases. The authors of “TRUST4RD: tool for reducing uncertainties in the evidence generation for specialised treatments for rare diseases” presented recommendations for reducing uncertainty in rare diseases. Their article is of great importance but unfortunately suffers from a lack of references to the wider uncertainty in Health Technology Assessment and research prioritisation literature and consequently fails to provide a trusted framework for decision-making in rare diseases. In this letter to the editor we critique the authors’ tool and provide pointers as to how their proposal can be strengthened. We present references to the literature, including our own tool for uncertainty assessment (TRUST; unrelated to the authors’ research), apply TRUST to two assessments of orphan drugs in rare diseases and provide a broader perspective on uncertainty and risk management in rare diseases, including a detailed research agenda.

## Background

In their position paper entitled “TRUST4RD: tool for reducing uncertainties in the evidence generation for specialised treatments for rare diseases”, Annemans & Makady present recommendations for reducing uncertainty in rare diseases. We very much welcomed this article, which recognises the importance of uncertainty and its implications for Health Technology Assessments (HTA) decision-making, with a particular focus on rare diseases. HTA in the area of rare diseases may suffer from more uncertainty than in other conditions and it is vital that this is dealt with transparently. This article therefore tackles an important topic and shows that more research is needed into the identification, assessment and management of uncertainty in general, as well as in rare diseases.

Our group of authors have developed a tool for identifying, assessing and reporting uncertainty, the TRansparent Uncertainty ASsessmenT (TRUST) tool [1], independently of the research presented by Annemans & Makady and published earlier in 2020. Based on our own research in this area, we think that the article could have benefitted from more detail, clarification and placement in the quickly developing literature in this field. Our aim of this letter to the editor is to provide a comprehensive summary of uncertainty assessment in Health Technology Assessment, with a focus on transferability to the setting of rare diseases. To do this, we critically review the TRUST4RD tool, make suggestions for improvement and reference the wider literature, we demonstrate the use of TRUST in two assessments of orphan drugs in rare diseases, and, finally, a present a broader perspective on uncertainty and risk management and propose a research agenda.

## TRUST4RD by Annemans & Makady: suggestions for improvement

Annemans & Makady set out broad considerations around reducing uncertainty in HTA of rare diseases. These considerations consist of three “building blocks”: 1) evidence gaps, 2) data sources and design, and 3) the presence of an iterative dialogue. The authors then move on to describe a process for finding a common solution. We present a critique as well as suggestions for improvement and references to the wider literature for each of these. It may be worth mentioning that the acronym TRUST4RD is not defined by Annemans & Makady.

For 1), the authors claim a new taxonomy of evidence gaps, distinguishing four main types of uncertainties important in rare diseases, according to which uncertainties should be identified early on in the HTA:

- uncertainties related to the size and characteristics of the population;
- uncertainties related to the natural history of the disease and its current management;
- uncertainties related to the new treatment;
- and uncertainties related to the health ecosystem.

We are concerned that these four uncertain aspects of an HTA may unnecessarily restrict stakeholders in their uncertainty identification process. Whilst we acknowledge that these four aspects may indeed be important in rare diseases, we find these to lack specificity. It is unclear to us and may be unclear to other users, whether certain aspects of a HTA are included in these four aspects. For example, health-related quality of life estimates can be a significant source of uncertainty in rare diseases. It could be argued that “uncertainties related to the natural history of the disease and its current management” include health-related quality of life aspects. If this were the case, then it is difficult to see how these four aspects are a reduction of all aspects in a HTA. Furthermore, we would argue to the contrary, that, in order to ensure explicit and transparent presentation of uncertainties, a less condensed view of aspects would be beneficial. Assessments of rare diseases are likely heterogeneous and sources of uncertainty could affect all aspects, so we urge caution against blinding ourselves to aspects with a lower prevalence. The TRUST tool, which was based on a review of the literature and HTA report guidelines and validated using stakeholder interviews, workshops and case studies, distinguishes between the following aspects of an assessment that may be subject to uncertainty: context or scope—including population, intervention, comparator, outcomes, time horizon and perspective; selection of evidence; model structure; model inputs—including transition probabilities, time-to-event, or accuracy estimates; relative effectiveness estimates; adverse events; utilities; resource use and costs; and model implementation. We consider that, even in non-model-based assessments, these distinctions may be helpful and allow for a more comprehensive identification process and more specific assessment and reporting.

The claim to a taxonomy may furthermore be misplaced. Multiple taxonomies of uncertainty exist and they tend to have in common that aspects or locations (such as those proposed by the authors and the assessment aspects considered in TRUST as discussed in the previous paragraph) are only one dimension of uncertainty. The other dimensions include the nature of uncertainty, so whether it is epistemic or aleatory; and sources of uncertainty, sometimes also referred to as “levels” [2]. In our TRUST tool we synthesized the latter based on the literature [3–8] as follows: transparency issues, methodological issues, imprecision, indirectness and unavailability. These distinctions may help in study design as well as in making decisions about how to explore and analyse this uncertainty.

We agree with Annemans & Makady that not all uncertainties are worth researching. How to achieve research prioritisation within HTA, however, has been much debated in the past and methods are available to help with this, namely value of information analysis [4, 9–12]. The authors’ solution is multi-stakeholder involvement; and potential impact scoring of uncertainties. Unfortunately, no methods to derive such an impact score are provided in the article. A part of the TRUST tool enables researchers and analysts to assess the impact of uncertainties on outcomes. In interviews, meetings and case study applications at the Dutch Health Care Institute conducted for the development of TRUST, we found that numerically scoring uncertainties for their impact was not only difficult for stakeholders, it also resulted in substantial inter-rater variability and could evoke the impression of quantified uncertainty, whereas scores were really based on subjective perception. These impact scores were therefore abandoned. We think that, if such scores were to be introduced for HTAs, further research would need to examine how this could work in practice. However, based on our research findings, we consider the benefit of this to be questionable. Instead, in the TRUST tool we proposed a simple assessment of whether the impact would be “likely high”, “likely low”, or “likely no impact”. Annemans & Makady go on to propose “what-if” scenarios to explore the impact of uncertainties, which we consider important, too. However, other commonly used methods [4] such as one-way sensitivity analysis, scenario analyses including threshold analysis, probabilistic sensitivity analyses, model averaging [13] and value of information methods should not be disregarded.

For 2), the authors recommend that different study designs be explored and matched with the evidence gap. There is no specific guidance on exactly what study designs would be beneficial in what settings, although the authors do provide some helpful examples. We think that the TRUST tool’s different sources of uncertainty may be informative in devising study designs. Furthermore, we consider that the impact of uncertainties alone cannot dictate research priorities. As argued by many scholars, the cost of the research has to be weighed up against the value of research and this assessment should be undertaken for different research targets and study designs [14, 15]. Methods like value of information and real options analyses enable such assessments. There are, of course, caveats: not all assessments are based on models that include all uncertainties. Qualitative judgements will have to be made in practice, but we should be clear on the “gold standard”, being an open and transparent consideration of the value of reducing uncertainties and the costs, as well as practical and ethical concerns associated with the potential research [16].

Research should not be considered in isolation when addressing risk in HTA decision-making. Uncertainties that have an impact on the decision at the originally proposed price may no longer do so at a reduced price [17]. Furthermore, research can be coupled with other schemes that target an effective price reduction, such as in pay-for-performance schemes. Consideration of all Managed Entry Agreement options is therefore vital [18–20].

For 3), we cannot argue with the importance of an iterative dialogue and collaboration throughout the process. The authors recommend early dialogue between manufacturers and regulatory bodies and also advocate for the application of the principled compromise concept in finding a common solution to addressing uncertainties. We think this is an excellent suggestion and would like to see an application of this following a real-world prospective case study in the future. In the meantime, we suggest the use of TRUST for supporting decision-makers in setting up a reflexive assessment that allows for transparent debate of all uncertainties and their best possible management.

## Application of TRUST to assessments of orphan drugs for treatment of rare diseases

The TRUST tool (available at https://osf.io/8eay7/?) was filled in by one researcher involved in the assessment and the filled in tool was checked by a second researcher familiar with the case. The researchers discussed whether any particular challenges were encountered in using TRUST, that may have been caused by the rarity of disease, and any scope for further development of TRUST so that it can be used in rare diseases.

- Case 1: NICE appraisal “Fenfluramine for treating Dravet syndrome” (ongoing in 2020)

Fenfluramine was compared with standard of care for the treatment of patients with Dravet syndrome [21]. Dravet syndrome is a severe life-limiting form of epilepsy characterised by epileptic seizures as well as cognitive-behavioural impairment and motor disorders and affecting children and adults. This NICE appraisal suffered from uncertainty, even though the main trial was adequately powered. Most impactful uncertainty aspects were located in the context/scope, model structure, effectiveness, relative effectiveness estimates, and utilities (see Table 1 for more detail). Most uncertainties were not fully included in the model or explored in sensitivity analyses.

- Case 2: NICE appraisal "Sebelipase alfa for treating lysosomal acid lipase deficiency" (appeal ongoing in 2020)

**Table 1:** Application of TRUST in assessments of orphan drugs in rare diseases

NICE appraisal “Fenfluramine for treating Dravet syndrome” (ongoing in 2020)	NICE appraisal "Sebelipase alfa for treating lysosomal acid lipase deficiency" (appeal ongoing in 2020)
*Context/scope* The phase III fenfluramine trials targeted children or adolescents ≤ 18 years old, but the population considered in included children or adolescents that aged in adulthood as well as patients that initiated fenfluramine in adulthood. A relevant comparator was excluded	*Model structure* lack of clarity on natural history and health states patients transition to; important health states might have been left out. Model structure differs per treatment arm due to a lack of evidence
*Model structure* Once patients discontinued from treatment, they were assumed to revert to baseline seizure frequency and not to the placebo ‘on-treatment’ seizure frequency. Removing the presumed placebo effect for discontinued patients while not removing it for patients on treatment would likely result in an overestimated treatment effect for being on treatment versus patients that discontinued treatment	*Effectiveness* extremely small sample size for new intervention (n = 9). Only a surrogate, rather than a clinical, endpoint available. Methods for lifelong extrapolation of transition probabilities were not transparent
*Effectiveness* Patient profiles generated contained seemingly inconsistent/implausible patient profiles and the methods to extrapolate the estimated convulsive seizures frequency/free days were unclear	*Relative treatment effectiveness* historical control had small sample size (n = 36). Patient baseline characteristics may not have been comparable between the two arms
*Relative treatment effectiveness* **(**1) Treatment effectiveness was based on an indirect-treatment comparison of different subgroups (cannabidiol with clobazam vs. Fenfluramine with/without clobazam); (2) the company assumed that the relative treatment effect is constant and maintained over time while patients are on treatment; (3) the company assumed the same percentage reduction for convulsive seizure days as was estimated (based on the NMA) for convulsive seizure frequency; (4) it is assumed that mortality was linked to convulsive seizure frequency without sufficient evidence to support this	*Adverse events* were not included in the model, the observed adverse events were mainly allergic reactions. Given the small sample sizes it may be possible that adverse events were missed
*Utilities* Lack of methodological guidance on how to include carer utilities	*Utilities* there was a lack of clarity on why selected utility values were chosen and whether they were appropriate. Utilities for infants were based on assumptions

Sebelipase alfa was compared with best supportive care for treatment of patients wilt lysosomal acid lipase (LAL) deficiency [22]. LAL deficiency is an inherited autosomal recessive lysosomal storage disorder. Mutations in the lysosomal acid lipase gene result in deficiency of the LAL enzyme. This causes abnormal accumulation of lipids, mainly in the gastrointestinal, hepatic and cardiovascular systems. It causes gastrointestinal and liver problems including malabsorption, growth failure, profound weight loss, steatorrhoea (excretion of fat in stools) and hepatomegaly (enlarged liver). The median life expectancy of a baby with rapidly progressive LAL deficiency is 3.7 months, but onset can also occur later in life (median onset at five years of age). This assessment suffered from significant uncertainty. Most impactful uncertainty aspects were located in the estimation of treatment effectiveness; the natural history of the disease; relative effectiveness; adverse events and health-related quality of life (see Table 1). Most uncertainties were not fully included in the model or explored in sensitivity analyses.

## Conclusions from both cases

Both examples illustrate the high levels of uncertainty present in many assessments of orphan drugs in rare diseases. Especially the case of sebelipase alfa is representative of the difficulty of gathering evidence, even on the historical control or natural history of the disease, often encountered in rare diseases. Reaching a reimbursement decision under such circumstances is potentially associated with a significant risk. It has not become clear from filling in TRUST which research study design may present the most value or even value for money and should therefore be pursued. The researchers did not encounter any difficulties in filling in TRUST. The researchers thought that no uncertainties were left out although there may be some unknown unknowns [2]. In conclusion, TRUST seems to work well in identifying most impactful uncertainties also in assessments of orphan drugs in rare diseases. It offers a systematic and transparent overview of all uncertainties and an estimate of their impact. In order to identify the optimal further research study design, model improvements are indicated to be able to quantitatively explore the impact of uncertainties. Where this is not possible, results from TRUST can serve as a basis for deliberation on the most valuable research targets.

## Uncertainty management in rare diseases going forward

**Fig. 1:**
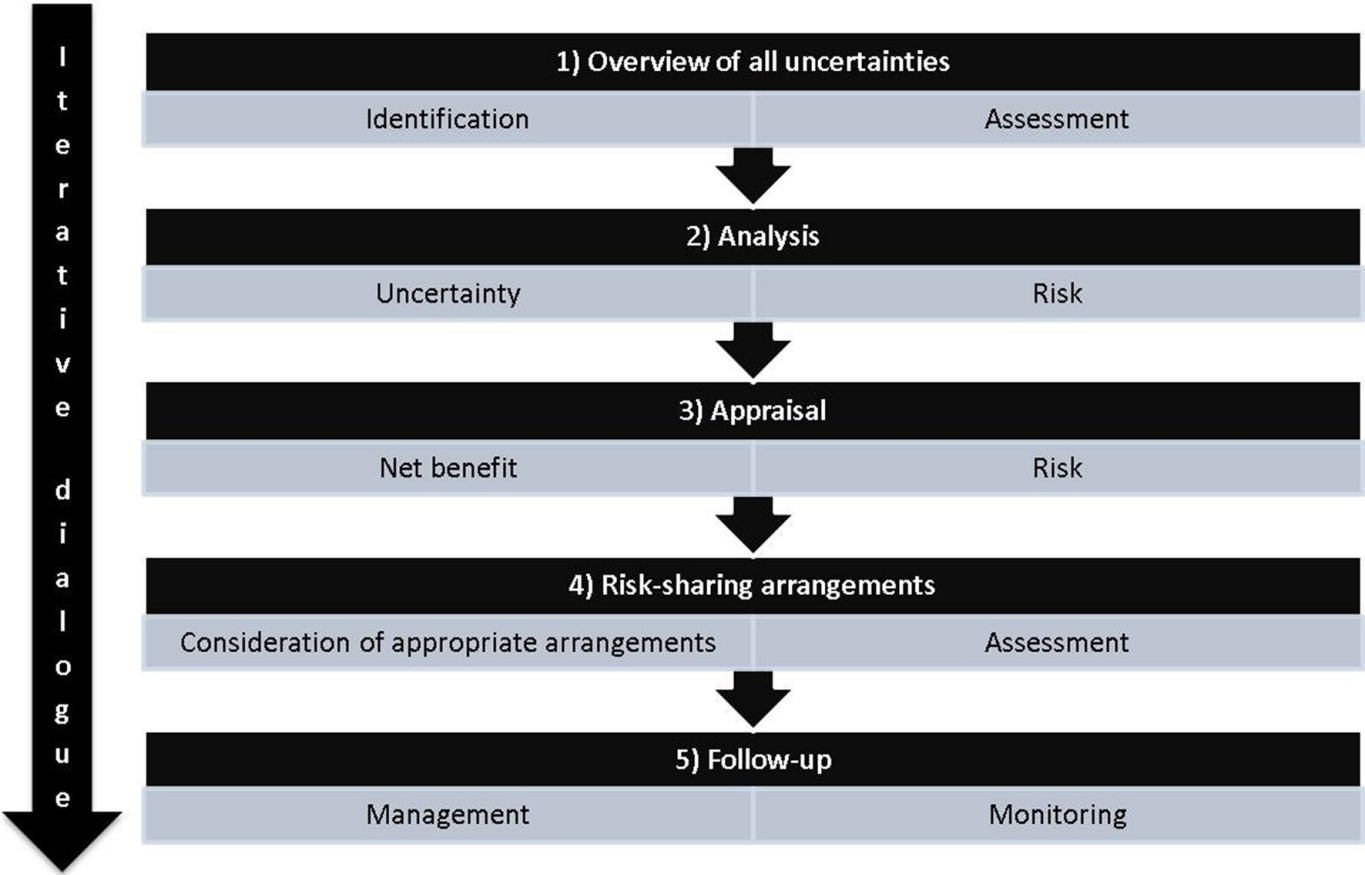
Five steps of uncertainty management in rare diseases

Assessments in rare diseases will always be subject to uncertainty. It is all the more important that uncertainty management receives due consideration. In this paper we showed that identification of uncertainties in rare diseases can be done systematically, transparently and comprehensively. However, there are challenges for the future. To provide a research agenda for this topic, we suggest breaking down the rather large topic of uncertainty management into five steps (Fig. 1).

1. A clear overview of uncertainties necessitates identification of all uncertainties relevant in a decision problem. Assessment of impact (based on quantitative analysis if available or subjective opinion) can help in prioritising those uncertainties that should be analysed further. This step can be aided by tools such as the TRUST tool [1].
2. Ideally, all uncertainties are quantified in a decision model and incorporated in the probabilistic analysis [4]. Uncertainties lead to a risk of making a “wrong” decision, such as making a recommendation for reimbursement, when the technology is, in fact, not cost effective, or vice versa. Risk is composed of the outcomes of a decision and the probability of occurrence [23]. Risk analysis can be performed using value of information methods [9, 24]. Quantitative analyses may not always be feasible due to time and resource constraints. In such cases, the assessment of impactful uncertainties may be used to inform the appraisal. Further research should be performed on exploring uncertainties that are difficult to quantify [25].
3. Two features are of interest in the appraisal for a reimbursement decision: the expected net benefit (or expected cost effectiveness) of the new intervention and risk [14, 20]. It depends on these two whether, and if so what type of, risk-sharing arrangements may be indicated. In orphan diseases, per patient risk is typically high. The risk of making a wrong decision for the whole health system or society, on the other hand, is probably often low due to the population being small. For example, even if a new technology turned out not to be cost-effective, cost to society would be limited (population risk). However, the uncertainty about adverse events may result in a high risk for each patient that receives the new treatment (per patient risk). Further research into risk appraisal in orphan diseases may be worthwhile.
4. Risk-sharing arrangements can be used to manage the risk. They are broadly composed of financial schemes (outcome-based or simple, not outcome-based) and data collection schemes (such as trials or collection of observational data [20]), or hybrid schemes that make use of both options. First, consideration has to be given to potentially appropriate risk-sharing arrangements. Risk-sharing arrangements can then be assessed quantitatively where possible, for example using the HTA risk analysis framework [17, 26], which requires value of information analysis [27–30]. Further research could inform a more qualitative approach to identifying research targets, study designs and negotiating an acceptable agreement with manufacturers to ensure appropriate pricing. Furthermore, it may be worth thinking about whether further data collection should not always be considered in the appraisal of orphan drugs.
5. Follow-up is important: new data may necessitate a reaction and this needs to be planned for. Management of any risk-sharing arrangements and planning of future re-appraisals as well as monitoring are crucial. It would be informative to study experiences from risk-sharing arrangements and how they have been managed.

As proposed by Annemans & Makady, an iterative dialogue amongst stakeholders with their multiple diverse perspectives is key. Ideally, this will be upheld throughout all the steps from early assessment to follow-up. Further research may focus on how this could be implemented in existing HTA processes.

## Conclusion

We provided a comprehensive summary of uncertainty assessment in Health Technology Assessment, with a focus on transferability to the setting of rare diseases. We made suggestions for improving TRUST4RD in the context of the rapidly emerging uncertainty literature. Application of the unrelated TRUST tool proved valuable for identifying, and assessing the impact of, uncertainties in two assessments of orphan drugs in rare diseases. We defined five steps to uncertainty management and proposed a research agenda specific to these in the field of rare diseases.

## Data Availability

Not applicable.

## References

[1] Grimm SE, Pouwels X, Ramaekers BLT, Wijnen B, Knies S, Grutters J (2020). Development and validation of the transparent uncertainty assessment (TRUST) tool for assessing uncertainties in health economic decision models. Pharmacoeconomics.

[2] Walker WE, Harremoes P, Rotmans J, Van der Sluijs JP, Van Asselt MBA, Janssen P (2003). Defining uncertainty: a conceptual basis for uncertainty management in model-based decision support. Integrated Assessment..

[3] van der Bles AM, van der Linden S, Freeman ALJ, Mitchell J, Galvao AB, Zaval L (2019). Communicating uncertainty about facts, numbers and science. R Soc Open Sci.

[4] Briggs AH, Weinstein MC, Fenwick EA, Karnon J, Sculpher MJ, Paltiel AD (2012). Model parameter estimation and uncertainty analysis: a report of the ISPOR-SMDM modeling good research practices task force working group-6. Med Decis Making.

[5] van Asselt MBA, Rotmans J (2002). Uncertainty in integrated assessment modelling: from positivism to pluralism. Climatic Change..

[6] Bouwknegt M, Havelaar A. Uncertainty assessment using the NUSAP approach: a case study on the EFoNAO tool. EFSA supporting publication. 2015;EN-663.

[7] Balshem H, Helfand M, Schunemann HJ, Oxman AD, Kunz R, Brozek J (2011). GRADE guidelines: 3. Rating the quality of evidence. J Clin Epidemiol.

[8] Guyatt GH, Oxman AD, Vist GE, Kunz R, Falck-Ytter Y, Alonso-Coello P (2008). GRADE: an emerging consensus on rating quality of evidence and strength of recommendations. Bmj.

[9] Rothery C, Strong M, Koffijberg HE, Basu A, Ghabri S, Knies S (2020). Value of information analytical methods: report 2 of the ISPOR value of information analysis emerging good practices task force. Value Health.

[10] Claxton K, Sculpher M, Drummond M (2002). A rational framework for decision making by the National Institute For Clinical Excellence (NICE). Lancet.

[11] Eckermann S, Willan AR (2007). Expected value of information and decision making in HTA. Health Econ.

[12] Minelli C, Baio G (2015). Value of Information: A Tool to Improve Research Prioritization and Reduce Waste. PLoS Med.

[13] Bojke L, Claxton K, Sculpher M, Palmer S (2009). Characterizing structural uncertainty in decision analytic models: a review and application of methods. Value Health.

[14] Chalkidou K, Lord J, Fischer A, Littlejohns P (2008). Evidence-based decision making: when should we wait for more information?. Health Aff (Millwood).

[15] Eckermann S, Karnon J, Willan AR (2010). The value of value of information: best informing research design and prioritization using current methods. Pharmacoeconomics.

[16] van der Wilt GJ, Grutters JPC, Maas A, Rolden HJA (2018). Combining value of information analysis and ethical argumentation in decisions on participation of vulnerable patients in clinical research. BMC Med Ethics.

[17] Grimm SE, Strong M, Brennan A, Wailoo AJ (2017). The HTA risk analysis chart: visualising the need for and potential value of managed entry agreements in health technology assessment. Pharmacoeconomics.

[18] Carlson JJ, Chen S, Garrison LP (2017). Performance-based risk-sharing arrangements: an updated international review. Pharmacoeconomics.

[19] Garrison LP, Towse A, Briggs A, de Pouvourville G, Grueger J, Mohr PE (2013). Performance-based risk-sharing arrangements-good practices for design, implementation, and evaluation: report of the ISPOR good practices for performance-based risk-sharing arrangements task force. Value Health.

[20] Walker S, Sculpher M, Claxton K, Palmer S (2012). Coverage with evidence development, only in research, risk sharing, or patient access scheme? A framework for coverage decisions. Value Health.

[21] NICE. Fenfluramine for treating Dravet syndrome [ID1109] 2020 [October 2020]. Available from: https://www.nice.org.uk/guidance/indevelopment/gid-ta10373.

[22] NICE. Lysosomal acid lipase deficiency - sebelipase alfa [ID737] 2020 [October 2020]. Available from: https://www.nice.org.uk/guidance/indevelopment/gid-lysosomalacidlipasedeficiencysebelipasealfaid737.

[23] Renn O (2008). Risk Governance: Coping with Uncertainty in a Complex World.

[24] Fenwick E, Steuten L, Knies S, Ghabri S, Basu A, Murray JF (2020). Value of information analysis for research decisions-an introduction: report 1 of the ISPOR value of information analysis emerging good practices task force. Value Health.

[25] Ghabri S, Cleemput I, Josselin JM (2018). Towards a new framework for addressing structural uncertainty in health technology assessment guidelines. Pharmacoeconomics.

[26] Grimm S, Strong M, Brennan A, Wailoo A (2016). Framework for analysing risk in health technology assessments and its application to managed entry agreements.

[27] Kunst N, Wilson ECF, Glynn D, Alarid-Escudero F, Baio G, Brennan A (2020). Computing the expected value of sample information efficiently: practical guidance and recommendations for four model-based methods. Value Health..

[28] Heath A, Kunst N, Jackson C, Strong M, Alarid-Escudero F, Goldhaber-Fiebert JD (2020). Calculating the expected value of sample information in practice: considerations from 3 Case Studies. Med Decis Making.

[29] Heath A, Manolopoulou I, Baio G (2017). A review of methods for analysis of the expected value of information. Med Decis Making.

[30] Wilson EC (2015). A practical guide to value of information analysis. Pharmacoeconomics.

