# Correction to: Building a trusted framework for uncertainty assessment in rare diseases: suggestions for improvement (Response to “TRUST4RD: tool for reducing uncertainties in the evidence generation for specialised treatments for rare diseases”)

**DOI:** 10.1186/s13023-021-01938-7

**Published:** 2021-07-19

**Authors:** Sabine E. Grimm, Xavier Pouwels, Bram L. T. Ramaekers, Ben Wijnen, Saskia Knies, Janneke Grutters, Manuela A. Joore

**Affiliations:** 1 Department of Clinical Epidemiology and Medical Technology Assessment, School for Public Health and Primary Care (CAPHRI), Maastricht University Medical Centre, P. Debyelaan 25, PO Box 5800, 6202 AZ Maastricht, Netherlands; 2 University of Twente, Hallenweg 5, 7522 NH Enschede, Netherlands; 3 Zorginstituut Nederland, Eekholt 4, 1112 XH Diemen, Netherlands; 4 Department for Health Evidence, Radboud University Medical Centre, Post 133, PO Box 9101, 6500 HB Nijmegen, Netherlands

## Correction to: Orphanet J Rare Dis (2021) 16:62 https://doi.org/10.1186/s13023-020-01666-4

Following the publication of the original article [1] it was brought to our attention that some of the authors are part of the Evidence Review Group for the England & Wales National Institute for Health and Care Excellence (NICE) and did not yet have permission to publish the case studies in this article, because final guidance has not been issued.

Any mentions to the case studies have, therefore, been removed from the original article, which has already been updated online.
